# An Olfactory Piriform Cortex‐Lateral Hypothalamus Neural Circuit Contributes to Linalool‐Induced Analgesia

**DOI:** 10.1002/cns.71072

**Published:** 2026-08-04

**Authors:** Yunhe Zhu, Jingping Sun, Shan Wang, Qidong Zhang, Jian Mao, Guobi Chai, Qingzhao Shi, Wu Fan, Jianping Xie

**Affiliations:** ^1^ College of Chemistry Zhengzhou University Zhengzhou Henan China; ^2^ Zhengzhou Tobacco Research Institute of CNTC Zhengzhou Henan China; ^3^ Beijing Life Science Academy Beijing China

**Keywords:** aromatherapy, lateral hypothalamus, linalool, neural circuit, pain, piriform cortex

## Abstract

**Aims:**

Aromatherapy‐mediated analgesia is widely applied, but the underlying neural mechanisms remain poorly understood. This study aimed to investigate the neurobiological basis underlying linalool‐induced attenuation of inflammatory pain.

**Methods:**

A complete Freund's adjuvant (CFA)‐induced hind‐paw inflammatory pain model was used to evaluate the antinociceptive effects of linalool odor exposure through mechanical and thermal nociceptive threshold tests, together with conditioned place preference. Viral tracing, in vivo fiber photometry, electrophysiological recordings, and chemogenetic or optogenetic manipulations were employed to identify and functionally characterize the neural circuits underlying linalool‐induced analgesia.

**Results:**

Linalool odor exposure increased mechanical and thermal nociceptive thresholds and induced pain relief‐associated place preference in CFA mice. Linalool exposure activated orexin A‐expressing neurons in the lateral hypothalamus (LH^orexin A^) and LH‐projecting glutamatergic neurons in the piriform cortex (Piri^Glu^‐LH). Electrophysiological recordings demonstrated functional excitatory monosynaptic connectivity from the Piri to the LH. Activation of LH‐projecting Piri^Glu^ neurons produced analgesic effects in CFA mice, whereas inhibition of this pathway attenuated linalool‐induced analgesia.

**Conclusion:**

Our study identified an olfactory cortex‐hypothalamic circuit contributing to linalool‐induced antinociceptive effects, providing mechanistic insight into olfactory modulation of inflammatory pain.

## Graphical abstract

Linalool odor exposure activates a glutamatergic projection from the piriform cortex to the lateral hypothalamus (Piri^Glu^‐LH), mediating descending inhibitory pain pathways. This demonstrates a novel neural mechanism for olfactory‐induced pain modulation and suggests potential therapeutic implications for pain management.

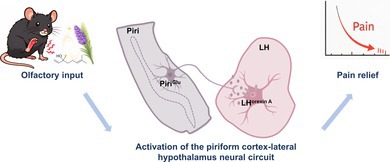

## 1 Introduction

Pain is a complex physiological and psychological experience arising from actual or potential tissue damage and is modulated by emotion, cognition, and memory [1, 2]. In recent years, various nonpharmacological approaches for pain modulation have been applied in clinical pain management, including olfactory, light, and auditory stimulation [3, 4, 5, 6]. Olfactory analgesia, which originates from aromatherapy, refers to the ability of odor stimuli to produce analgesic effects by modulating emotional states and the activity of the Pain Matrix of the brain [7, 8]. Clinical studies have shown that pleasant scents, such as lavender, vanilla, and banana, can reduce subjective pain intensity [9]. In particular, lavender essential oil has been widely used in clinical settings to alleviate inflammatory, cancer‐related, visceral, and neuropathic pain [10, 11]. Similar findings have been reported in rodents. Floral odors, such as phenylethanol and lavender essential oil, reduce nociceptive responses and suppress excessive neuronal activity in the trigeminal system [12]. Yang et al. [13] reported that inhalation of lavender essential oil reduces hyperactivity of glutamatergic neurons in the insular cortex, thereby alleviating inflammatory pain. However, studies investigating the neural mechanisms underlying olfactory analgesia remain limited.

The lateral hypothalamus (LH) functions as an integrative hub that receives inputs from feeding, emotional, and sensory systems [14, 15]. Increasing evidence indicates that the LH plays an important role in pain modulation [16]. In particular, Orexin A‐expressing neurons within the LH (LH^orexin A^) have been widely implicated in the regulation of nociception and are involved in several forms of nonpharmacological analgesia [15, 17]. Acute stress, such as restraint or forced swim stress, activates hypothalamic orexinergic neurons, which suppress nociceptive transmission through descending inhibitory pathways; accordingly, disruption of orexin signaling attenuates stress‐induced analgesia [18]. Similarly, electroacupuncture and voluntary exercise have been shown to reduce pain sensitivity and induce pain‐relief‐associated cue preference in mice, accompanied by activation of LH^orexin A^ neurons. Pharmacological blockade of orexin receptors using the antagonist SB‐334867 significantly diminishes both the analgesic effect and the associated positive behavioral responses [19, 20]. However, whether LH^orexin A^ neurons participate in odor‐induced analgesia remains unknown.

The LH exhibits significant neural responses to odor stimuli, which is structurally supported by its extensive projection inputs from olfactory‐related brain regions [21]. Retrograde tracing studies using wheat germ lectin‐horseradish peroxidase (WGA‐HRP) or cholera toxin B subunit (CTB) injections into the LH revealed labeled neurons in olfactory‐related regions, including the piriform cortex (Piri), anterior olfactory nucleus (AON), olfactory tubercle (OT), and amygdala [22, 23]. Among these olfactory regions, the Piri serves as a major center for higher‐order olfactory processing and integration of odor information [24]. Recent studies by Dong et al., using anterograde viral tracing in Fos^TRAP2^ mice, further demonstrated that the LH receives projection fibers from glutamatergic neurons in the Piri [25]. However, some studies have suggested that olfactory information may also reach the LH through pathways independent of the Piri or OT circuits [26]. These discrepancies may reflect state‐ or task‐dependent recruitment of distinct olfactory pathways, as well as differences in species and experimental paradigms across studies. Despite accumulating anatomical evidence supporting olfactory‐LH connectivity, the functional relationship between the LH and the Piri has not been systematically investigated, and whether such connections contribute to odor‐induced analgesia remains largely unexplored.

In this study, using viral tracing, optogenetics manipulation, and electrophysiological recordings, we identified a monosynaptic glutamatergic projection from the piriform cortex to the lateral hypothalamus (Piri^Glu^‐LH), which is activated during linalool‐induced olfactory analgesia. Chemogenetic activation of the LH‐projecting Piri^Glu^ neurons recapitulates the antinociceptive effects of linalool inhalation in CFA mice. Conversely, inhibition of this pathway abolishes linalool‐induced antinociception. These findings represent an advancement in understanding the neural circuit mechanisms underlying odor‐induced analgesia, underscoring the therapeutic potential of leveraging specific odorants for pain management.

## 2 Results

### 2.1 Exposure to Linalool Odor Induced Significant Analgesic Effects

**FIGURE 1:**
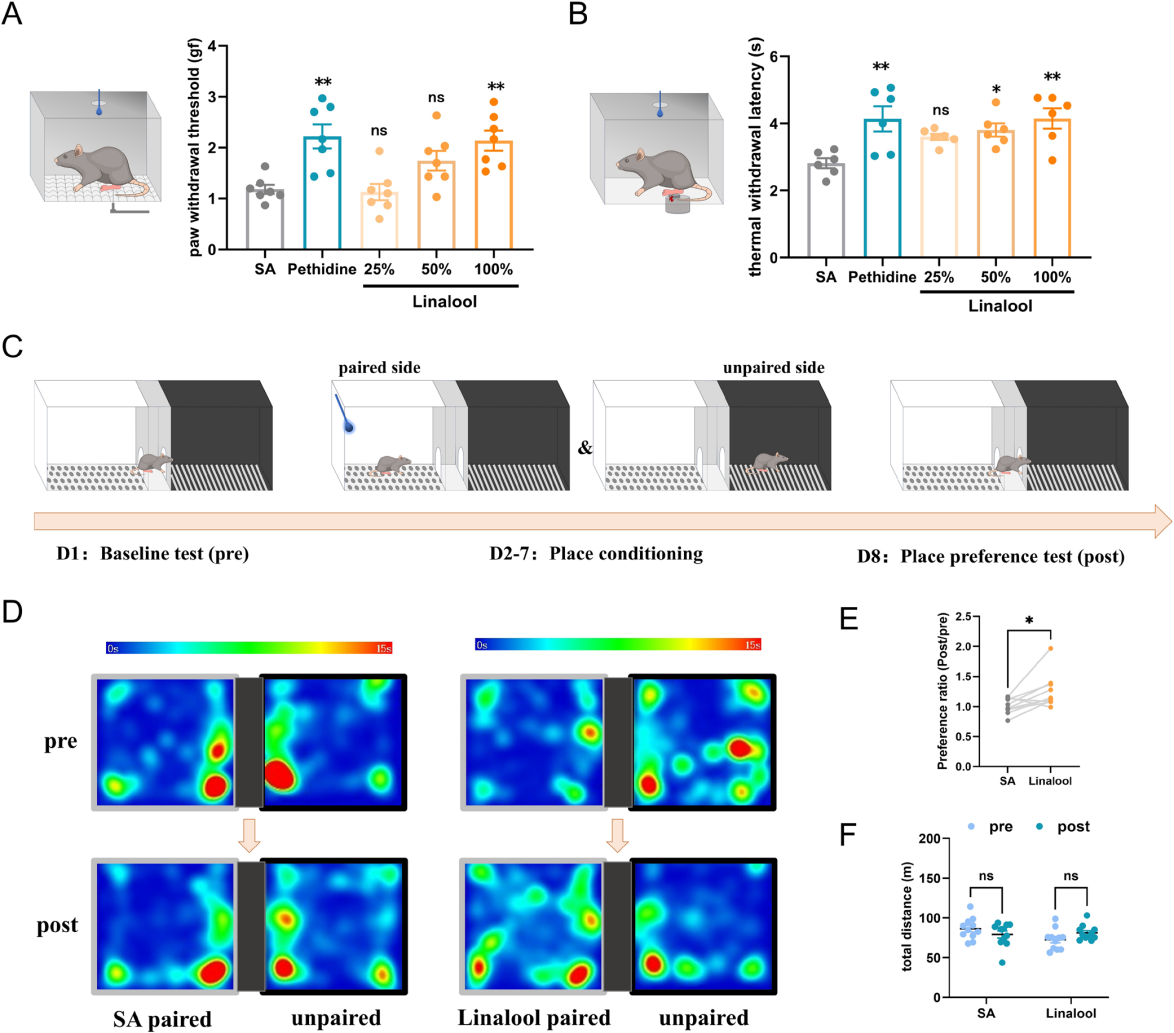
Linalool exposure induced analgesic effects. (A) Effects of different concentrations of linalool odor exposure on mechanical pain sensitivity in CFA‐treated male mice (*n* = 7, *F*_(4,30)_ = 7.981, *p* = 0.0002). (B) Effects of different concentrations of linalool odor exposure on thermal pain sensitivity in CFA‐treated male mice (*n* = 6; *F*_(4,25)_ = 4.948, *p* = 0.0045). (C) Schematic of experimental timeline for the CPP test. (D) Representative heatmaps of travel trajectory of CFA mice treated with SA or linalool odor exposure in the CPP test. (E) Summarized data for preference ratio for the odor‐paired side in CFA mice from the indicated group (*n* = 10, *t*_9_ = 2.877, *p* = 0.0183). (F) Total distance traveled (SA, *n* = 10; Linalool, *n* = 10; *F*_(1,40)_ = 4.118, *p* = 0.0491) in the CPP test for CFA‐treated mice exposed to SA or linalool. All data are expressed as the mean ± SEM. *p < 0.05; **p < 0.01; ns, not significant.

Complete Freund's adjuvant (CFA) was injected into the hindpaw to establish a mouse model of inflammatory pain [3]. Mechanical and thermal nociceptive thresholds were assessed using electronic von Frey and plantar test, respectively (Figure S1A,B). To identify odorants with potential analgesic effects, we screened a panel of structurally diverse odorants in CFA mice. Different odorants produced distinct effects on pain‐related behaviors, among which linalool, the main compound of lavender oil, produced the most robust analgesic effect, comparable to the positive control pethidine (0.2 mg/10 g) (Figure S1C,D). CFA mice were then exposed to different concentrations of linalool, which increased mechanical and thermal nociceptive thresholds in a dose‐dependent manner, with the strongest analgesic effect observed at 100% concentration (Figure 1A,B). To further determine whether this effect depends on intact olfactory function, we employed a methimazole‐induced olfactory epithelium lesion model [27]. Olfactory deprivation markedly attenuated the analgesic effects of linalool exposure, indicating that intact olfactory processing is required for the full expression of linalool‐induced analgesia (Figure S2A). In addition, serum IL‐1β, TNF‐α, and corticosterone levels remained unchanged following linalool odor exposure (Figure S2B–D), suggesting that the observed behavioral effects are unlikely to be mediated by the suppression of peripheral inflammatory responses or by stress‐induced endocrine responses.

Given that pain relief is considered rewarding [28], we employed a conditioned place preference (CPP) test to evaluate the linalool‐induced preference in CFA‐treated mice (Figure 1C). The results showed that a significant increase in the time CFA‐treated mice spent on the linalool‐paired side, without a significant change in total locomotor activity (Figure 1D–F). In non‐pain‐sensitive mice, linalool exposure did not induce a preference for the paired side (Figure S3), suggesting that the rewarding effect of linalool odor stimulation arises from the process of pain relief rather than an intrinsic preference for the odor. Notably, similar analgesic effects were also observed in female CFA mice (Figure S4). To reduce animal use and improve consistency, subsequent experiments in this study were conducted using male mice.

We further evaluated the potential effects of linalool exposure on anxiety‐like behaviors in CFA mice using the open field and elevated zero maze tests (Figure S5). After 10 min of linalool odor exposure, the percentage of time spent in the center area, percentage of time in light area, and light area entries was significantly increased, suggesting an alleviation of anxiety‐like behavior. No motor deficits were detected, as evidenced by the lack of difference in total distance traveled between saline‐ and linalool‐exposure mice. Notably, similar anxiolytic effects of inhaled linalool have also been reported in non‐pain‐related anxiety models, suggesting that its anxiolytic action is unlikely to be merely secondary to pain relief, but may instead represent an additional olfactory‐driven behavioral effect occurring in parallel with analgesia [29, 30].

### 2.2 The LH^Orexin^ ^A^ Are Involved in Linalool‐Induced Analgesic Activity

**FIGURE 2:**
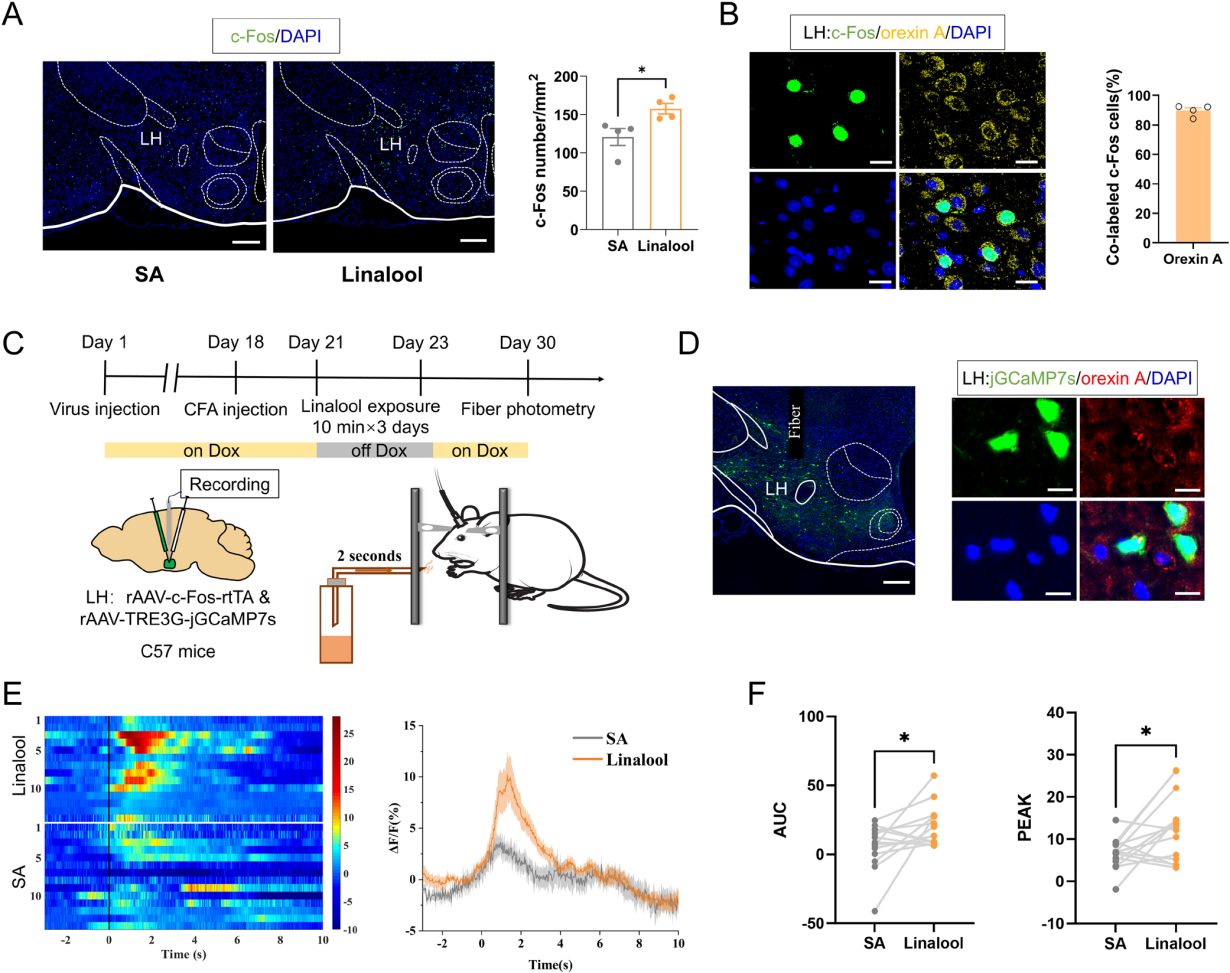
Linalool odor exposure enhances the activity of LH^orexin A^ neurons. (A) Representative images and quantitative analysis of c‐Fos^+^ neurons in the LH following linalool exposure (scale bar: 200 μm; *n* = 4 mice, 4 sections per mouse, *t*_6_ = 2.820, *p* = 0.0304). (B) Representative immunofluorescence images and statistical proportion of c‐Fos^+^ neurons co‐localized with orexin A antibody in LH (scale bar: 20 μm; *n* = 4 mice). (C) Experimental timeline illustrating viral injection, Dox regulation, and fiber photometry recording, together with schematic diagrams of viral delivery and optical fiber implantation in the LH, as well as the head‐fixed linalool odor exposure paradigm. (D) Histological verification of jGCaMP7s expression in the LH and representative immunofluorescence images showing co‐localization of jGCaMP7s‐positive neurons with orexin A antibody (scale bars: 200 μm and 10 μm). (E) Heatmap and the averaged Δ*F*/*F* traces showing rapid increases in LH^orexin A^ Ca^2+^ signals induced by linalool exposure. (F) Quantification of calcium responses including the area under the Δ*F*/*F* curve and peak Δ*F*/*F* responses (*n* = 14; AUC *t*_13_ = 2.366, *p* = 0.0342; PEAK *t*_13_ = 2.627, *p* = 0.0209). All data are expressed as the mean ± SEM. *p < 0.05.

The roles of the hypothalamus, particularly the lateral hypothalamus (LH), in pain regulation have been extensively studied [31]. We found that chemogenetic activation of LH neurons significantly attenuated both mechanical and thermal hypersensitivity, producing an analgesic effect that closely mirrored the behavioral improvement elicited by linalool odor exposure (Figure S6). To determine whether linalool could activate orexin A‐expressing neurons in the LH, mice were randomly assigned to the Linalool or salt solution (SA) groups and placed individually in the test chamber for 30 min. Mice were then returned to their home cages and perfused 1 h later for immunostaining of the immediate‐early gene product c‐Fos. Results revealed a significant increase in c‐Fos^+^ cells in the LH following linalool odor exposure, and approximately 90% of these c‐Fos^+^ neurons were co‐labeled with orexin A, suggesting that LH orexinergic neurons represent the primary population responsive to linalool stimulation (Figure 2A,B).

We employed a Tet‐off system to selectively label LH^orexin A^ neurons activated during linalool exposure and monitored their activity using fiber photometry (Figure 2C). rAAV‐c‐Fos‐rtTA and rAAV‐TRE3G‐jGCaMP7s were co‐injected into the LH of C57BL/6, which were then maintained on a doxycycline‐containing diet (on Dox) to inhibit rtTA binding to the TRE promoter and prevent nonspecific expression of jGCaMP7s. After Dox withdrawn (off Dox), mice were exposed to linalool odor stimulation for 10 min daily, during which rtTA was expressed only in LH neurons activated by linalool and expressing c‐Fos, thereby driving TRE3G promoter‐mediated jGCaMP7s expression. Immunofluorescence analysis showed substantial overlap between jGCaMP7s‐positive neurons and c‐Fos immunoreactivity, indicating that the Tet‐off system effectively labeled a population of linalool‐responsive neurons (Figure S7). In addition, LH neurons expressing the calcium indicator jGCaMP7s were highly co‐labeled with orexin A immunoreactivity (Figure 2D). During recording, mice were kept awake and head‐fixed to ensure consistent and effective odor delivery. Odor stimuli were delivered for 2 s with an inter‐trial interval of 60 s. Compared with SA, linalool odor stimulation induced a significant increase in calcium signals immediately after odor delivery (Figure 2E). To quantitatively assess the extent of the linalool odor calcium responses, we analyzed calcium signals within 6 s after odor delivery using the area under the curve (AUC) and peak value as indicators. As shown in Figure 2F, both the AUC and peak values of calcium signals were significantly higher during linalool stimulation than SA. These findings demonstrate that LH^orexin A^ neurons activity is closely correlated with linalool odor stimuli.

**FIGURE 3:**
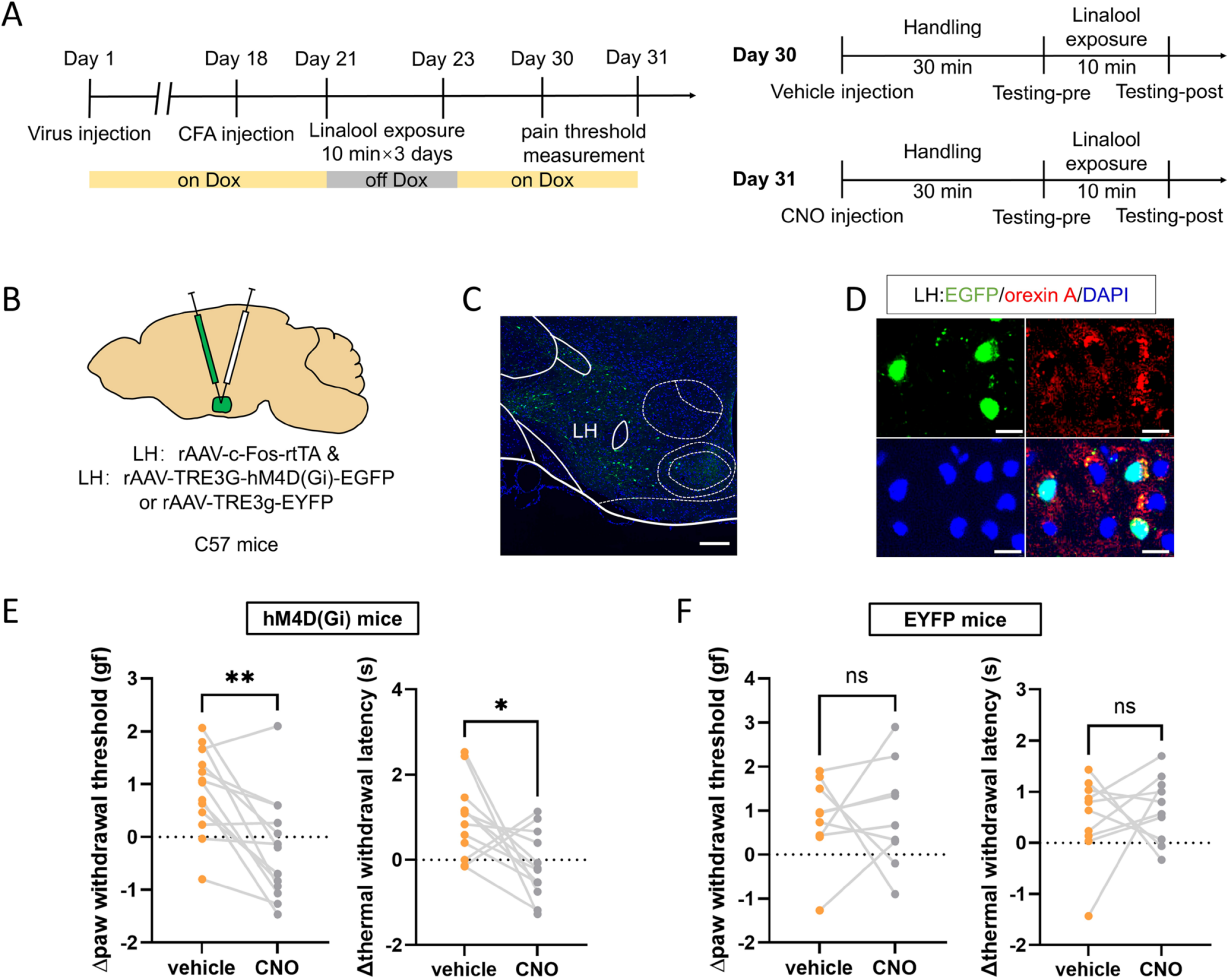
Chemogenetic inhibition of LH^orexin A^ neurons attenuates linalool‐induced analgesia. (A) Experimental timeline illustrating virus injection, Tet‐off manipulation, and behavioral testing. (B) Schematic illustrating viral strategies for activity‐dependent labeling and chemogenetic manipulation of LH^orexin A^ neurons. (C) Representative image showing viral expression within the LH (scale bar: 200 μm). (D) Immunofluorescence images demonstrating colocalization of Tet‐off‐labeled EGFP‐positive neurons with orexin A immunoreactivity in the LH (scale bar: 10 μm). (E) In hM4D(Gi)‐expressing mice, CNO administration (1 mg/kg, i.p.) significantly reduced the linalool‐induced increase in mechanical paw‐withdrawal threshold (left; *n* = 13, *t*_12_ = 3.994, *p* = 0.0018) and thermal withdrawal latency (right; *n* = 12, *t*_11_ = 2.557, *p* = 0.0257). (F) In EYFP control mice, CNO administration did not affect linalool‐induced changes in mechanical withdrawal threshold (left; *n* = 9, *t*_8_ = 0.1501, *p* = 0.8844) or thermal withdrawal latency (right; *n* = 10, *t*_9_ = 0.4550, *p* = 0.6599). All data are expressed as the mean ± SEM. *p < 0.05; **p < 0.01; ns., not significant.

To further test the functional role of LH^orexin A^ neurons in linalool‐induced analgesia, we selectively inhibited this neuronal population using a chemogenetic approach. Also using the Tet‐off system, LH^orexin A^ neurons were activity‐dependently labeled to express either the inhibitory DREADD hM4D(Gi) or the control protein EYFP, with immunofluorescence confirming high colocalization with orexin A (Figure 3A–D). After viral expression, mice received CNO (1 mg/kg) or vehicle, followed by baseline pain threshold measurements and subsequent exposure to linalool odor. Analgesic effects were quantified as the change in mechanical and thermal pain thresholds before and after odor exposure (Figure 3A). In hM4D(Gi)‐expressing mice, CNO administration significantly attenuated linalool‐induced increases in both mechanical withdrawal thresholds and thermal withdrawal latencies (Figure 3E), whereas no significant effects were observed in EYFP‐expressing control mice (Figure 3F). These results indicated that LH^orexin A^ neuronal activity is required for linalool‐induced analgesic effects.

### 2.3 Piri^Glu^ Is an Upstream Cortex Region Conveying Olfactory Signal to LH^orexin^ ^A^

**FIGURE 4:**
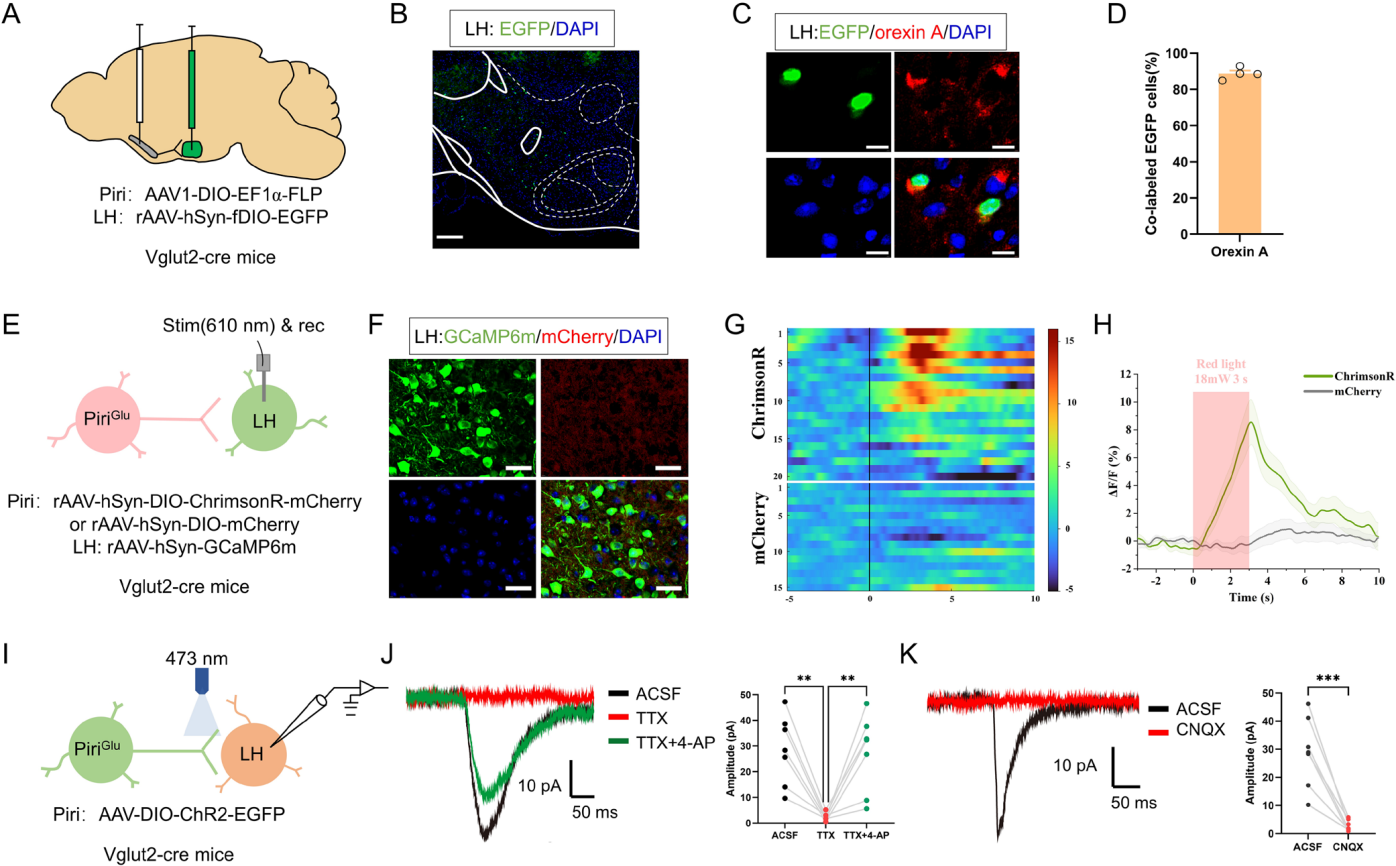
Piri^Glu^ neurons project to LH neurons. (A) Schematic illustration of the viral strategy used for anterograde transsynaptic labeling of LH neurons receiving from Piri^Glu^. (B) Representative images showing transsynaptically labeled LH neurons receiving inputs from Piri^Glu^ (scale bar: 200 μm). (C and D) Representative images showing orexin A immunoreactivity in transsynaptically labeled LH neurons receiving inputs from Piri^Glu^ and summarized quantification data (scale bar: 10 μm; *n* = 4 mice). (E) Schematic of virus injection and fiber photometry stimulation & recordings. (F) representative coronal images showing the expression of ChrimsonR‐mCherry and GCaMP6m in the LH (scale bar: 30 μm). (G and H) The heatmap and mean data showing that Ca^2+^ signals were rapidly increased following optical stimulations in the LH. (I) Schematic illustrating optogenetic stimulation coupled with electrophysiological recording. (J) Representative traces and summary data of optogenetically evoked postsynaptic currents recorded from LH neurons under ACSF, in the presence of TTX (1 μM), or TTX plus 4‐AP (50 μM) (*n* = 7 cells from four mice; *F*_(2,18)_ = 11.19, *p* = 0.0007). (K) Representative traces and summary data of optogenetically evoked postsynaptic currents recorded from LH neurons under ACSF and in the presence of the AMPA receptor antagonist CNQX (20 μM) (*n* = 7 cells from four mice; *t*_6_ = 5.986, *p* = 0.0010). All data are expressed as the mean ± SEM. **p < 0.01; ***p < 0.001.

To identify the presynaptic inputs to LH neurons, retroAAV‐hSyn‐EGFP was injected into the LH, revealing that Layer 3 of the olfaction‐related piriform cortex (Piri) is one of the upstream regions projecting to the LH (Figure S8). Furthermore, retroAAV‐TRE3G‐Cre was injected into the LH and rAAV‐c‐Fos‐DIO‐rtTA‐EGFP into the Piri of C57BL/6 mice to characterize the neuronal subtypes of Piri neurons projecting to the LH that are activated by linalool stimulation in CFA‐treated mice. Immunofluorescence staining showed that these EGFP^+^ neurons were co‐localization with glutamatergic markers (Figure S9). Anterograde tracing was performed by injecting rAAV‐hSyn‐DIO‐mGFP‐T2A‐Synaptophysin‐mRuby into the Piri of vglut2‐Cre mice, revealing mRuby‐positive terminals originating from Piri^Glu^ neurons in the LH (Figure S10). Finally, specific labeling of Piri^Glu^‐LH projection neurons was achieved by injecting rAAV1‐DIO‐FLP into the Piri of the vglut2‐cre mice while simultaneously injecting rAAV‐fDIO‐EGFP into the LH (Figure 4A). AAV1 possesses anterograde transsynaptic properties, FLP recombinase was transferred to downstream LH neurons receiving inputs from Piri^Glu^ neurons, thereby inducing FLP‐dependent EGFP expression in these postsynaptic neurons. Immunofluorescence analysis demonstrated that a substantial proportion of these transsynaptically labeled LH neurons exhibited orexin A immunoreactivity (Figure 4B–D). Together, these retrograde and anterograde tracing results provide convergent anatomical evidence supporting the existence of a Piri^Glu^‐LH^orexin A^ pathway.

To explore the functional connectivity of the Piri^Glu^‐LH circuit, we injected rAAV‐hSyn‐DIO‐ChrimsonR‐mCherry or rAAV‐hSyn‐DIO‐mCherry into the Piri of vglut2‐cre mice, along with rAAV‐hSyn‐GCaMP6m into the LH. Fibers were inserted into the LH when AAVs fully expressed to activate Piri^Glu^ terminals in the LH and simultaneously recorded calcium activity of LH neurons. Upon photo‐stimulation of Piri^Glu^ terminals, LH exhibited significantly elevated calcium fluorescence compared to control (mCherry‐only) mice, demonstrating a functional connectivity of the Piri^Glu^‐LH circuit (Figure 4E–H). Whole‐cell recordings combined with optogenetics in brain slices showed that brief light stimulation of ChR2‐containing Piri^Glu^ terminals elicited excitatory postsynaptic currents (EPSCs) in the postsynaptic LH (Figure 4I). The optically evoked EPSCs were abolished by the AMPA receptor antagonist CNQX (Figure 4J). Furthermore, these light‐evoked EPSCs were completely blocked by TTX but were rescued by 4‐AP, indicating that Piri^Glu^ neurons form monosynaptic excitatory synapses onto the LH neurons (Figure 4K).

### 2.4 Linalool Exposure Activated Piri^Glu^ ‐LH Circuit

**FIGURE 5:**
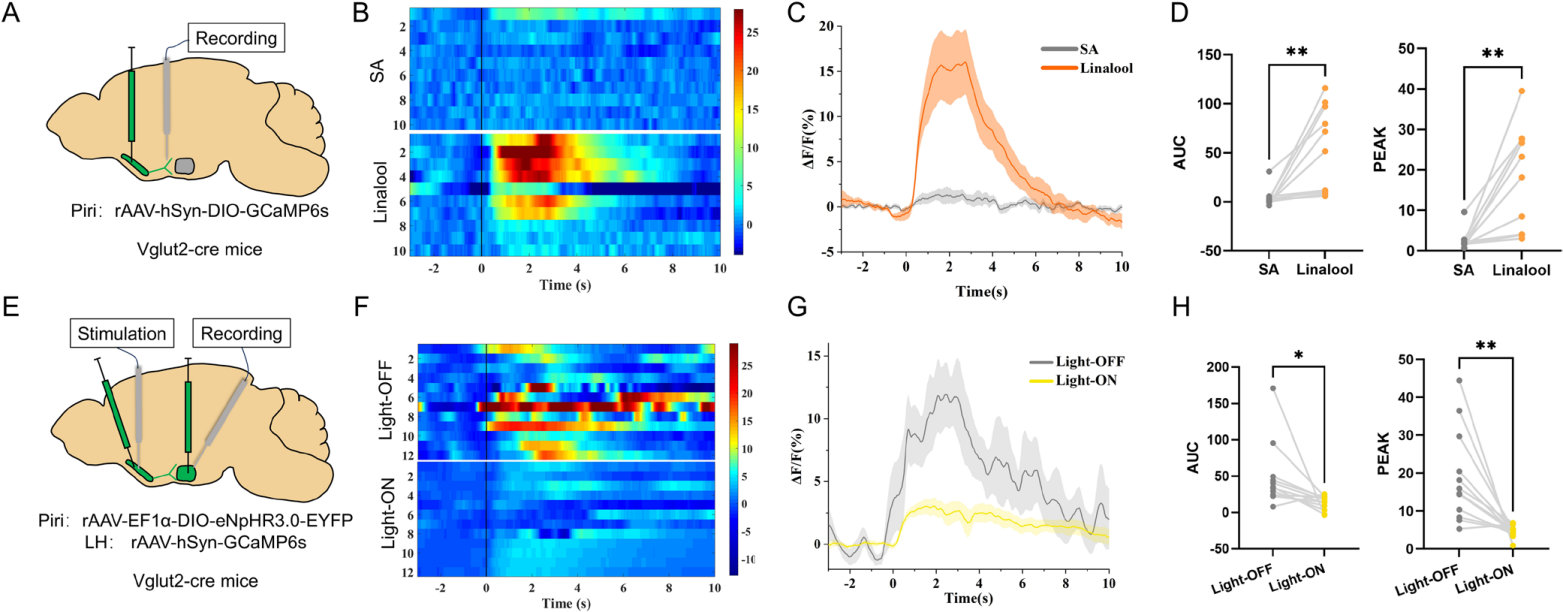
Linalool exposure activated Piri^Glu^‐LH circuit. (A) Schematic of viral injection for fiber photometry recording of calcium activity in Piri^Glu^ neurons projecting to the LH. (B and C) The heatmap (B) and mean data (C) showing that Ca^2+^ signals were rapidly increased by linalool exposure. The colored bar on the right indicated ∆*F*/*F* (%). (D) Summarized data including AUC (left, *n* = 10, *t*_9_ = 3.688, *p* = 0.0050) and PEAK (right, *n* = 10, *t*_9_ = 4.011, *p* = 0.0031) of mean data in (B) and (C). (E) Schematic of viral injection for optogenetics and fiber photometry strategy to assess LH neuronal responses under Piri^Glu^ inhibition. (F–G) The heatmap (F) and mean data (G) showing that optogenetic inhibition of Piri^Glu^ neurons markedly reduced LH Ca^2+^ responses to linalool exposure. The colored bar on the right indicated ∆*F*/*F* (%). (H) Summarized data including AUC (left, *n* = 12, *t*_11_ = 2.455, *p* = 0.0319) and PEAK (right, *n* = 12, *t*_11_ = 3.681, *p* = 0.0036) of mean data in (F) and (G). All data are expressed as the mean ± SEM. *p < 0.05; **p < 0.01.

To investigate the real‐time activity changes of Piri^Glu^ neurons in response to linalool odor stimulation, we injected a calcium indicator virus (rAAV‐hSyn‐DIO‐GCaMP6s) into the Piri of vglut2‐cre mice (Figure 5A). Head‐fixed mice were then exposed to 2 s odor pulses as described above. Following linalool exposure, fluorescence intensity in LH‐projecting Piri^Glu^ neurons increased rapidly (Figure 5B,C), with both the AUC and PEAK values within 6 s significantly higher than those in the saline control group (Figure 5D). Furthermore, to assess whether LH neuronal responses depend on Piri^Glu^ activity, we injected rAAV‐EF1α‐DIO‐eNpHR3.0‐EYFP into the Piri and rAAV‐hSyn‐GCaMP6s into the LH of vglut2‐cre mice (Figure 5E). When Piri^Glu^ neuronal activity was optogenetically inhibited, linalool‐induced activation of LH was markedly reduced (Figure 5F–H). Together, these results indicate that linalool robustly activates the Piri^Glu^‐LH circuit, and that Piri^Glu^ neuronal activity is essential for mediating LH responses to linalool stimulation.

### 2.5 The Piri^Glu^ ‐LH^orexin^ ^A^ Pathway Contributes to Analgesic Effect Induced by Linalool Odor Exposure

**FIGURE 6:**
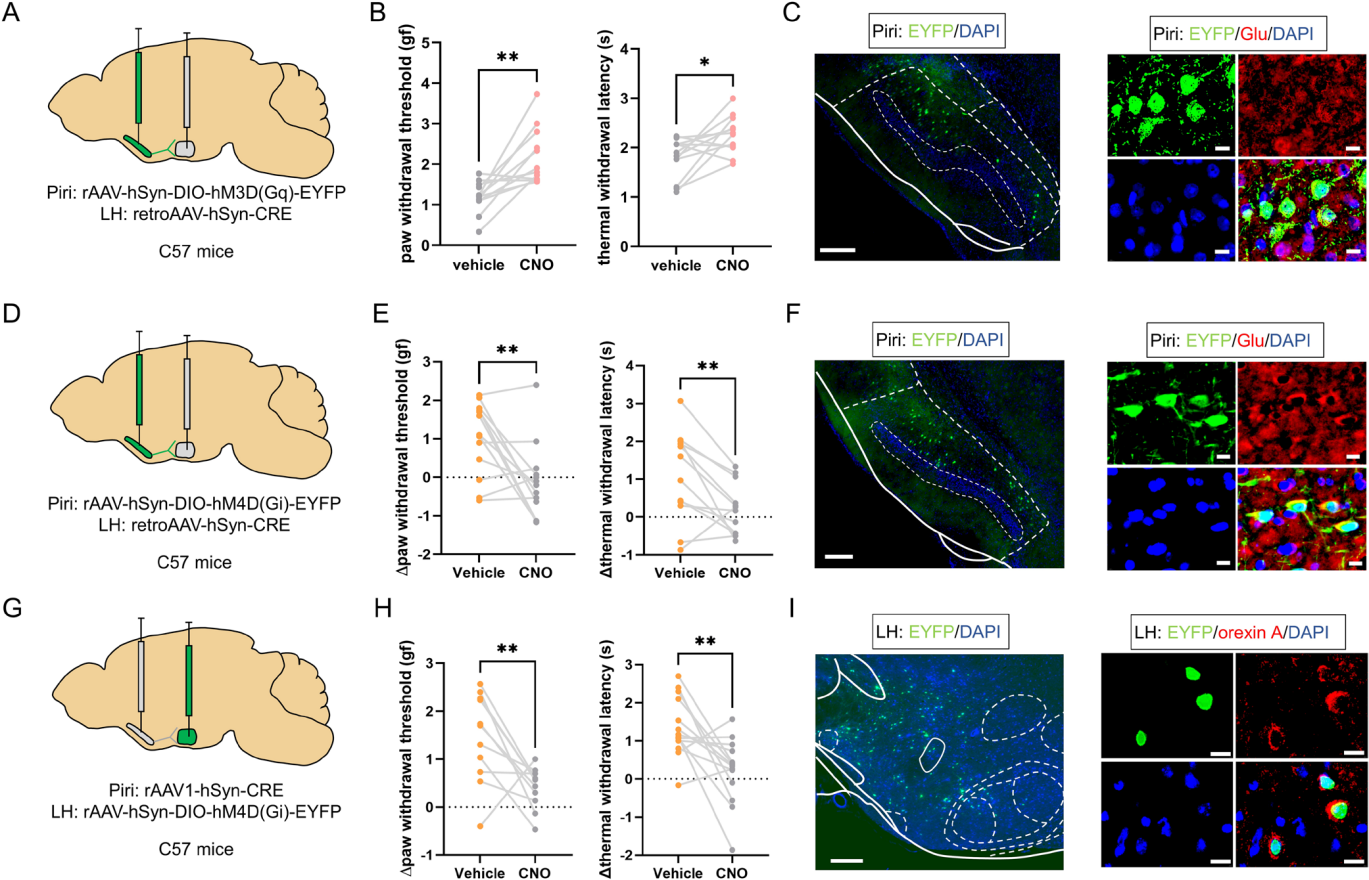
The Piri^Glu^‐LH^orexin A^ projection mediates the linalool‐induced analgesia. (A) Schematic of chemogenetic activation strategy of Piri^Glu^ neurons projecting to the LH. (B) The mechanical nociceptive threshold (*n* = 12; *t*_11_ = 4.182, *p* = 0.0015) and thermal nociceptive threshold (*n* = 12; *t*_11_ = 2.886, *p* = 0.0148) following CNO or vehicle in hM3D(Gq) expressing mice. (C) Representative images validating hM3D(Gq) expression in glutamatergic neurons of Piri (scale bars: 200 μm and 10 μm). (D) Schematic of the chemogenetic inhibition strategy of Piri^Glu^ neurons projecting to the LH. (E) The mechanical paw‐withdrawal threshold (*n* = 13, *t*_12_ = 3.190, *p* = 0.0078) and thermal withdrawal latency (*n* = 12, *t*_11_ = 3.115, *p* = 0.0098) following CNO or vehicle in hM4D(Gi) expressing mice. (F) Representative images validating hM4D(Gi) expression in glutamatergic neurons of Piri (scale bars: 200 μm and 10 μm). (G) Schematic of the chemogenetic inhibition strategy targeting LH^orexin A^ neurons receiving inputs from Piri. (H) The mechanical paw‐withdrawal threshold (*n* = 11; *t*_10_ = 3.207, *p* = 0.0094) and thermal withdrawal latency (*n* = 14; *t*_13_ = 3.439, *p* = 0.0044) following CNO or vehicle in hM4D(Gi) expressing mice. (I) Representative images validating hM4D(Gi) expression in LH orexin A‐positive cells (scale bars: 200 μm and 10 μm). All data are expressed as the mean ± SEM. *p < 0.05; **p < 0.01; ns., not significant.

To assess whether activation of the Piri^Glu^‐LH pathway contributes to analgesia, the Piri was transduced with rAAV‐hSyn‐DIO‐hM3D(Gq)‐EYFP, while retroAAV‐hSyn‐Cre was injected into the LH to achieve pathway‐specific targeting. EYFP fluorescence confirmed successful expression of the excitatory DREADD in glutamatergic Piri neurons projecting to the LH. Systemic administration of CNO significantly increased paw withdrawal thresholds and prolonged withdrawal latencies compared with vehicle‐treated controls, indicating that selective activation of the Piri^Glu^‐LH circuit produces significant analgesic effects (Figure 6A–C).

We examined whether the activity of the Piri^Glu^‐LH is required for linalool‐induced analgesia in CFA mice. Specifically, hM4D(Gi) was selectively expressed in Piri^Glu^ neurons projecting to the LH by injecting rAAV‐hSyn‐DIO‐hM4D(Gi)‐EYFP into the Piri and retroAAV‐hSyn‐CRE‐mCherry into the LH. Piri^Glu^ neurons projecting to the LH were inhibited via intraperitoneal injection of CNO 30 min before the behavioral test. To exclude potential off‐target effects of CNO, corresponding EYFP control experiments were performed using the same experimental procedures (Figure S11A,B). Results showed that, compared with vehicle controls, CNO‐mediated inhibition of Piri^Glu^‐LH neurons significantly attenuated the linalool‐induced increase in nociceptive thresholds, whereas CNO administration in EYFP control mice did not affect nociceptive behaviors. Histological analysis confirmed accurate and selective expression of hM4D(Gi) or EYFP in Piri^Glu^‐LH neurons (Figure 6D–F).

In addition, we selectively inhibited LH^orexin A^ neurons receiving Piri inputs in CFA mice. Circuit‐specific inhibition significantly reduced the linalool‐induced increases in paw withdrawal threshold and thermal withdrawal latency. Parallel EYFP control experiments demonstrated that CNO alone did not alter nociceptive responses under the same conditions (Figure S11C,D). Histological analyses confirmed accurate viral expression and colocalization with LH^orexin A^ neurons receiving Piri inputs (Figure 6G–I). Taken together, these findings demonstrated that inhibition of the Piri^Glu^‐LH^orexin A^ circuit effectively abolished linalool‐induced analgesia in CFA mice, revealing that neuronal activity within this pathway is essential for mediating olfactory‐driven pain relief.

## 3 Discussion

Compared with conventional pharmacological analgesia strategies, olfactory stimulation‐owing to its non‐invasive nature, ease of administration, high patient compliance, and minimal side effects‐has emerged as an increasingly important direction in non‐pharmacological pain research [26, 27]. Linalool is the major bioactive constituent of lavender essential oil, which is one of the most widely used and extensively studied essential oils and has demonstrated consistent analgesic efficacy in surgical, cancer‐related, and visceral pain [12, 28, 29, 30]. However, the neural mechanisms by which linalool‐induced signals are processed in the brain to regulate pain perception remain poorly understood. In the present study, a glutamatergic projection from the piriform cortex to the lateral hypothalamus was identified, which was activated in association with linalool‐induced analgesia. Selective manipulation of this pathway either recapitulated or abolished the analgesic effects of odor exposure. All data supporting the findings of this study are present in the Table S1.

Anxiety is a prominent psychological comorbidity in chronic pain and can exacerbate pain symptoms, potentially creating a vicious cycle between pain and anxiety [16]. Clinical studies indicate that 60%–93% of patients with persistent pain experience comorbid anxiety, which is associated with increased pain severity and poorer clinical outcomes [32]. Our behavioral analyses showed that linalool exposure significantly reduced anxiety‐like behaviors in CFA mice, consistent with clinical studies concurrent reductions in pain and anxiety following odor‐based interventions [33, 34]. In addition to its anxiolytic effects, linalool exposure induced CPP in CFA mice, suggesting that odor stimulation may influence affective and motivational processes in addition to sensory pain processing. Notably, relief of ongoing pain can itself generate positive affective states and CPP [28]. Conversely, the anxiolytic effects of inhaled linalool have also been reported in pain‐independent experimental models, indicating that its emotional and preference‐related effects are not necessarily secondary to its analgesic effects [29, 30]. We cannot exclude the possibility that modulation of emotional and motivational processes contributes, at least in part, to the behavioral outcomes observed here. Future studies specifically designed to separate the sensory‐discriminative and affective‐motivational dimensions of pain will be necessary to clarify their respective contributions to linalool‐induced pain relief.

The Piri is a higher‐order olfactory center that receives diffuse and overlapping olfactory bulb projections, thereby enabling highly convergent sensory integration [35]. Notably, odor identity can be encoded by the activity of only a few hundred Piri neurons [36]. Glutamatergic pyramidal neurons, which are predominantly located in Layers 2 and 3 of the Piri and serve as the principal output neurons, are highly dependent on olfactory input, as evidenced by their selective degeneration following olfactory bulb ablation [37, 38]. Piri^Glu^ exhibit enlarged dendritic spines, enhanced excitatory synaptic transmission, and increased neuronal excitability during associative learning processes involving odor stimuli [39]. In addition, the Piri distributes olfactory information to multiple higher‐order brain regions through parallel projection pathways, thereby influencing diverse physiological and behavioral functions [40]. Previous studies have demonstrated that patterned stimulation of the Piri can directly induce long‐term potentiation and long‐term depression in the dentate gyrus and alter hippocampal gene expression profiles [41]. Yang et al. [13] reported that glutamatergic neurons in the anterior Piri regulate inflammatory pain via an insular inhibitory interneuron‐dependent pathway, and that activation of this Piri^Glu^‐insula circuit is essential for the analgesic effects of lavender essential oil exposure. Our findings extend previous observations by identifying linalool as a principal analgesic component of lavender essential oil and revealing a Piri^Glu^‐LH neural pathway that mediates its analgesic effects. Unlike the delayed analgesic effects induced by 7 days of lavender essential oil exposure, the rapid analgesia observed after 10 min of linalool exposure suggests that the Piri^Glu^‐LH pathway may modulate pain perception before potential longer‐term inflammatory or plasticity‐related mechanisms become engaged [42]. This difference may also explain why the downstream targets identified in the two studies differ, highlighting the possibility that distinct Piri output pathways may contribute to odor‐induced analgesia at different temporal scales.

Although orexinergic neurons have cell bodies anatomically restricted to the LH, they exert widespread influence on nociceptive processing across multiple levels of the pain through extensive projections to multiple brain regions and the spinal cord [43]. For example, local administration of orexin A into the rostral ventromedial medulla increases the firing rate of pain‐inhibitory neurons and significantly reduces formalin‐induced nocifensive behaviors [44]. In addition, activation of the LH orexinergic projection to the nucleus accumbens (NAc) induces CPP associated with pain relief, whereas blockade of orexin 1 receptor signaling in the NAc abolishes this effect [45]. Therefore, identifying the precise downstream targets of LH orexinergic neurons and determining which neuronal populations ultimately mediate nociceptive modulation across hierarchical levels, including the sensory‐discriminative and affective‐motivational dimensions of pain, represents important directions for future investigation.

Several limitations of the present study warrant consideration. First, whether the Piri‐LH neural circuit underlying linalool‐induced analgesia in mice is conserved and functionally relevant in humans remains unknown [46]. Second, although similar analgesic effects were observed in both male and female mice, most mechanistic experiments were performed in a CFA‐induced inflammatory pain model. Therefore, it remains unclear whether the identified circuit contributes to other pain modalities, such as neuropathic or cancer‐related pain.

In conclusion, our study highlights the critical role of the Piri^Glu^‐LH neural circuit in mediating linalool odor‐induced relief of inflammatory pain. Given the global prevalence of chronic pain, our research supports an olfaction‐based strategy for pain relief, which may complement existing therapeutic approaches.

## 4 Materials and Methods

### 4.1 Chemical Reagents and Viral Vectors

Linalool with purity over 98% was purchased from Macklin Biochemical Co. Ltd. (Shanghai, China). The Complete Freund's adjuvant (CFA) was purchased from Sigma‐Aldrich (Germany). Isoflurane was obtained from RWD Life Science Co. Ltd. (Shenzhen, China). Meperidine hydrochloride injection was purchased from Qinghai Pharmaceutical Factory Co. Ltd. Clozapine‐N‐oxide (CNO) was obtained from Widing Biotech Co. Ltd. (China). Primary antibodies included anti‐c‐Fos (ab222699; Abcam), anti‐VGLUT2 (MAB5504, Sigma‐Aldrich), and anti‐orexin A (MAB763; Sigma‐Aldrich). Fluorophore‐conjugated secondary antibodies were Goat Anti‐Mouse IgG (Alexa Fluor 647, ab150115; Abcam) and Donkey Anti‐Rabbit IgG (Alexa Fluor 488, ab150073; Abcam). The mouse corticosterone ELISA kit was purchased from Cusabio Bioengineering Co. Ltd. (Wuhan, China).

rAAV‐hSyn‐GCaMP6m (5.10E+12 vg/mL, BC‐0078) and rAAV‐hSyn‐DIO‐GCaMP6s (5.11E+12 vg/mL, BC‐0238) produced by Brain Case Biotech Ltd. (Wuhan, China). retroAAV‐hSyn‐EGFP (5.13E+12 vg/mL, PT‐1990), rAAV‐hSyn‐DIO‐Synaptophysin‐mRuby (5.11E+12 vg/mL, PT‐1244), retroAAV‐TRE3G (5.64E+12 vg/mL, PT‐0244), rAAV‐cfos‐DIO‐rtTA‐EGFP (6.10E+12 vg/mL, PT‐13724), rAAV‐hSyn‐DIO‐ChrimsonR‐mCherry (5.04E+12 vg/mL, PT‐1374), rAAV‐EF1α‐DIO‐ChR2‐mCherry (5.11E+12 vg/mL, PT‐0002), rAAV‐EF1α‐DIO‐eNpHR3.0‐EYFP (5.18E+12 vg/mL, PT‐0006), retroAAV‐hSyn‐CRE (6.00E+12 vg/mL, PT‐0407), rAAV‐hSyn‐DIO‐EYFP (6.09E+12 vg/mL, PT‐0114), and rAAV‐hSyn‐DIO‐hM4D(Gi)‐EYFP (5.62E+12 vg/mL, PT‐0344) were purchased from BrainVTA Co. Ltd. (Wuhan, China).

### 4.2 Animals

Wild‐type mice of C57BL/6J genetic background were purchased from Henan Huaxing Experimental Animal Center. Vglut2‐Cre transgenic mice were purchased from Jiangsu Nanjing Jinzhihe Biotechnology Co. Ltd. A temperature‐controlled facility with a 12:12 h light/dark cycle (lights on at 9:00 a.m.) was used to house the experimental animals.

### 4.3 Stereotaxic Surgery

The anesthesia machine, stereotaxic frame, and micro‐infusing pump used in stereotaxic surgeries were all purchased from RWD Life Science Co. Ltd. (Shenzhen, China). Mice were deeply anesthetized using isoflurane with oxygen (3% for inducing; 1.5%–2% for maintenance) and mounted on a stereotaxic frame. A small craniotomy above the target brain region was performed using a dental drill, and the skull was carefully removed. The eyes of the mice were kept moist using ophthalmic ointment throughout the surgery. Virus was infused into the target areas a fine glass micropipette with a tip diameter of 10–15 μm. The speed and volume of the injection were controlled using a micro‐infusing pump. The pipette was left in the place for an additional 10 min after the end of the injection and then withdrawn slowly to avoid back‐flow of the virus.

Viral injections were targeted into the following coordinates: LH (AP: −1.6 mm; ML: −1.15 mm; DV: −5.15 mm) and Piri (AP: 0.25 mm; ML: −2.8 mm; DV: −5.2 mm). Experiments were performed 3–6 weeks after virus injection. Optical fiber implants were placed 200 μm above the injection site for fiber photometry and optogenetics and were fixed to the skull with dental cement. Viral injection sites and the position of fiber implants in each mouse were confirmed histologically after the termination of the experiments. Only those animals with correct virus expression or fiber implantation were included for further analysis.

### 4.4 Behavioral Tests

#### 4.4.1 Complete Freund's Adjuvant Injection

Inflammatory pain was induced by injecting 20 μL of the CFA into the plantar surface of the left hindpaw of each mouse under isoflurane anesthesia. The induce persistent inflammatory pain, a second injection of the CFA (10 μL) was administered 7 days after the first injection.

#### 4.4.2 Assessment of Mechanical Allodynia and Thermal Hyperalgesia

Behavioral assessment procedures were conducted as described in previous publications [3]. Animals were habituated in perforated, transparent Plexiglas cubicles (5 cm by 8.5 cm by 6 cm) for 30 min before the experiment to become quiet and to allow testing.

Mechanical allodynia was conducted using an electronic von Frey instrument (38450; Ugo Basile, Italy) and according to prior reports. The paw withdrawal thresholds of mice were determined as the maximum force to elicit paw withdrawal. Three measurements were obtained at every time point and averaged for each test.

Thermal hyperalgesia was assessed using the plantar test apparatus (37570; Ugo Basile, Italy). The heat source was positioned under the plantar surface of the hind paw and activated with a radiant light beam, the intensity of which was chosen in preliminary studies to give baseline latencies from 6 to 7 s in control mice. The mean paw withdrawal latencies from hind paws were determined from the average of three separate trials.

#### 4.4.3 Conditioned Place Preference (CPP) Test

A two‐chamber apparatus (20 × 20 × 20 cm each) connected by a central corridor (8 × 20 × 20 cm) was used to examine odor‐induced place preference. On Day‐1, mice were allowed to freely explore the apparatus for 15 min without odor stimulation. The time spent in each chamber on the Day‐1 was recorded, and the chamber with less time spent was designated as the odor‐stimulation side. On Days‐2, 4, and 6, mice were allowed to move freely only in the odor‐stimulated compartment, while on Days‐3, 5, and 7, they were confined to the non‐stimulated compartment. Each pairing session lasted for 1 h. On the Day‐8, a 15‐min preference test was performed, during which mice freely explored the apparatus without odor stimulation. Mice movements trajectories and the time spent in each chamber were analyzed in real time using VisuTrack software. The preference ratio was calculated as the time spent in the stimulation‐paired chamber on the Day‐8 divided by the corresponding time on Day‐1 [3].

#### 4.4.4 Elevated Zero Maze Test

The elevated zero maze consisted of a circular track, 6 cm in width, 50 cm in outer diameter, and elevated 55 cm above the floor. The maze was divided into four equal areas, consisting of two opposing open areas and two opposing closed areas (enclosed by 15 cm‐high walls). Each mouse was individually placed in an open area and allowed to explore freely for 5 min. The percentage of time spent in the open area and the number of entries into open area were used to assess anxiety‐like behavior, while total distance traveled in the maze was used as a measure of locomotor activities [47]. The maze was cleaned with 75% ethanol between trials. Animal travel trajectories were video‐recorded and analyzed offline using ANY‐maze software (Stoelting Co.).

#### 4.4.5 Open Field Test

The open field chamber was made of gray acrylic sheet (40 × 40 × 40 cm) and divided into a central field (20 × 20 cm) and a peripheral field. Mice were placed in the center of the chamber and allowed to move freely for 5 min. The animal movement was tracked by an overhead mounted camera interfaced to a computer running the anymaze software. The open field was cleaned with 75% ethanol between each trial. Total distance moved in the field was measured to assess the locomotor activities. For probing the anxiety state, we analyzed the percentage of the time spent in the center field as described previously [48].

### 4.5 Odor Stimuli

For odor exposure in behavioral and c‐Fos immunostaining experiments, procedures were adapted from previously published procedures with some modifications [49]. It was performed in the cubicles where pain thresholds were measured. Cotton balls containing 20 μL of linalool were placed up the center of Plexiglas cubicles during testing. All mice were kept approximately 10 cm away from the odor source, ensuring no physical contact with the cotton balls. For behavioral testing involving both saline and linalool exposure in the same mouse, experiments were conducted in a fixed order: saline exposure was performed first, followed by linalool exposure. To prevent odor cross‐contamination, different exposure sessions were conducted on separate days.

For fiber photometry experiments, odor exposure was delivered using an Olfactometer (OM‐16E; RWD Life Science Co. Ltd., Shenzhen) Odor parameters were set as follows: dilution flow, 1500 sccm; delivery time, 2 s; delivery flow, 500 sccm; and inter‐trial interval, 60 s.

### 4.6 Fiber Photometry and Optogenetic Manipulation

The activity responses of LH neurons during linalool odor exposure and Piri photostimulation were monitored using fiber photometry (Thinker Tech Nanjing Bioscience Inc.). Briefly, a mono fiber optic patch cord connected to the fiber photometry system and was attached to the implanted fiber optic cannula using a ceramic sleeve and black heat‐shrinkable tubes. To record GCaMP6m fluorescence signals, the 470 nm wavelength was measured before and during linalool odor exposure. Furthermore, 410 nm LED light was recorded as an isosbestic control channel. Fluorescence change (∆*F*/*F*) was assessed as follows: ∆*F*/*F*_0_ = *F*(*t*)–*F*_0_(*t*)/*F*_0_(*t*). The baseline was defined as the signals recorded 3 s before odor stimulus. After analysis, the fiber‐tip locations in each mouse were histologically examined.

For ChrimsonR photostimulation of Piri^Glu^, 635 nm light laser (10‐ms, 20 Hz, with a 3‐s light‐on period) was delivered via an optic cable. Laser power was 18 mW measured at the tip of the fiber, which was implanted 0.3 mm above the targeted nucleus.

For eNpHR3.0 photostimulation of Piri^Glu^, 589 nm light laser (10‐ms, 20 Hz, with a 2‐s light‐on period) was delivered via an optic cable. Laser power was 16 mW measured at the tip of the fiber, which was implanted 0.3 mm above the targeted nucleus.

### 4.7 Activity‐Dependent Neuron Labeling

Twenty‐one days after viral injection, DOX was replaced the DOX diet (100 mg/kg) with regular chow for 48 h to activate the activity‐dependent labeling system. Mice were then exposed to linalool odor to induce activity‐driven EGFP expression.

### 4.8 Chemogenetic Manipulations

For in vivo chemogenetic manipulations, mice were randomly assigned to receive an intraperitoneal injection of CNO (1 mg/kg, dissolved in 5% DMSO) or an equivalent volume of vehicle (5% DMSO). Behavioral tests were performed 30 min post‐injection.

### 4.9 Slice Electrophysiology

Whole‐cell patch‐clamp recordings were performed in acute brain slices from mice that had been stereotaxically injected with AAV‐EF1α‐DIO‐hChR2‐mCherry into the Piri. Slice preparation and electrophysiological recording procedures followed the previous publications.

Mice were deeply anesthetized with isoflurane and transcardially perfused with ice‐cold cutting solution saturated with 95% O_2_/5% CO_2_. The cutting solution contained (in mM): 93 N‐methyl‐d‐glucamine (NMDG), 93 HCl, 2.5 KCl, 1.2 NaH_2_PO_4_, 30 NaHCO_3_, 25 D‐glucose, 20 HEPES, 5 Naascorbate, 2 thiourea, 3 Na‐pyruvate, 10 MgSO_4_, and 0.5 CaCl_2_; pH was adjusted to 7.35 with NMDG or HCl. The brain was carefully removed and sectioned using a vibratome (VT1200S; Leica Inc.). Coronal slices (250 μm) containing the left LH were prepared and initially recovered in NMDG cutting solution at 35°C for 10–13 min. Slices were then transferred to oxygenated artificial cerebrospinal fluid (ACSF) containing (in mM): 126 NaCl, 2.5 KCl, 0.5 CaCl_2_, 2 MgCl_2_, 26 NaHCO_3_, 1.25 NaH_2_PO_4_, and 10 glucose, and maintained at 25°C for at least 1 h before electrophysiological recordings.

Slices were transferred to a recording chamber and carbogen‐saturated ACSF was perfused constantly at a flow rate of 2–3 mL/min at room temperature. Neurons from left LH were identified by their location and morphology. For light‐evoked currents, pipettes were filled with a solution containing (in mM): 135 K‐gluconate, 5 KCl, 0.5 CaCl_2_, 10 HEPES, 2 Mg‐ATP, 0.1 GTP, and 5 EGTA (pH adjusted to 7.3 with KOH), and the osmolarity being 300 mOsm. Photostimulation (15 mW) was delivered through an optical fiber placed near the slice and connected to an intelligent optogenetic system. Photostimulation was triggered by TTL signals from Clampex software. All signals were acquired with a MultiClamp 700B amplifier (Molecular Devices), filtered at 2 kHz, and sampled at 10 kHz with a Digidata 1440A interface using Clampex 10.4 (Molecular Devices). Data were accepted when series resistance fluctuated within 20% of initial values.

### 4.10 Immunohistochemistry and Imaging

The mice were anesthetized with pentobarbital (20 mg/kg, i.p.) and transcardially perfused with 0.9% saline, followed by 4% PFA. The brain was carefully removed and post‐fixed in 4% PFA at least 24 h. Following dehydration in a 30% (w/v) sucrose solution, coronal sections (40 μm) were prepared using a cryostat (Leica CM1950, Germany).

For immunohistochemistry, these slices were first incubated in 0.3% (v/v) Triton X‐100 for 15 min, followed by blocking of non‐specific reactions with 10% donkey serum for 1 h at room temperature. Then, these slices were incubated with anti‐c‐fos (ab222699; Abcam), anti‐vglut2 (MAB5504; Sigma‐Aldrich), and anti‐orexin A (MAB763; Sigma‐Aldrich) diluted in blocking solution (1:500, 0.3% Triton X‐100, 10% donkey serum in PBS) at 4°C for 24 h. After washing with PBS (3 × 5 min), these slices were incubated with the fluorophore‐conjugated secondary antibodies (Goat Anti‐Mouse (Alexa Fluor 647, ab 150,115) or Donkey Anti‐Rabbit (Alexa Fluor 488, ab150073)) for 2 h at room temperature. Finally, these slices were incubated with DAPI (P36935, Thermo fisher scientific) for 5 min and then washed with PBS three times and mounted for imaging.

All fluorescence images, including the verification of virus expression and fiber implantation, were acquired on Pannoramic MIDI and Pannoramic DESK. Imaging settings were kept consistent for all groups. All brain sections were aligned to a standard brain atlas (Paxinos and Franklins's the Mouse Brain in Stereotaxic Coordinates·Fifth Edition) using ImageJ and Adobe Illustrator to determine anatomical boundaries. Cell counting within each region was performed blindly using ImageJ.

### 4.11 Statistical Analysis

The data obtained from mice with missed injections or optical fiber placement were excluded from further analysis by experimenters who were blinded to the experimental conditions. Data describe biological replicates, and the sample size for each experiment is indicated in the corresponding figure legend. All data in this study are presented as the mean ± SEM. GraphPad Prism 8 (Graph Pad Software Inc.) was used for analyses and graphing. An unpaired or paired two‐tailed Student's *t*‐test was conducted for the statistical comparisons of data between two groups. One‐ or two‐way analysis of variance (ANOVA), which was followed by Dunnett's or Šídák's post hoc test for multiple comparisons, was conducted for statistical evaluation of data among more than two groups. The significant levels are indicated as *p < 0.05, **p < 0.01, ***p < 0.001.

## Funding

This study was supported by National Natural Science Foundation of China (32072344 and 32130083), Scientific and Technological Project of CNTC (110202102001 and 110202403006), and Scientific Research Program of Innovation Platform in State Tobacco Monopoly Administration (402021AWCX06).

## Ethics Statement

All animal experimental protocols conformed to the Guidelines for the Care and Use of Laboratory Animals and were granted by the Life Science Ethics Review Committee of Zhengzhou University (ZZU‐LAC20240228(02)).

## Conflicts of Interest

The authors declare no conflicts of interest.

## Supporting information


**Figure S1:** Validation of the CFA‐induced inflammatory pain model and screening of odorant‐induced analgesic effects. (A) CFA‐induced changes in pain thresholds based on Electronic von Frey tests (saline, *n* = 7, CFA, *n* = 8, *F*_(3,52)_ = 7.792, *p* = 0.0002). (B) CFA‐induced changes in pain thresholds based on Hargreaves tests (B, saline, *n* = 8, CFA, *n* = 8, *F*_(3,56)_ = 13.19, *p* < 0.0001). (C) Changes in mechanical pain threshold in CFA mice after exposure to different odors (*n* = 6). (D) Changes in thermal pain threshold in CFA mice after exposure to different odors (*n* = 7).
**Figure S2:** Effects of linalool exposure on corticosterone levels and olfactory‐dependent analgesia in CFA mice. (A) Effects of olfactory deprivation on linalool‐induced changes in mechanical paw withdrawal threshold (left, *n* = 7, *t*_6_ = 5.299, **p = 0.0018) and thermal withdrawal latency (right, *n* = 9, *t*_8_ = 2.833, *p = 0.0220). (B) Comparison of serum IL‐1β levels among treatment groups in CFA mice (*F*_(2,18)_ = 0.1433, *p* = 0.8675). (C) Comparison of serum TNF‐α levels among treatment groups in CFA mice (*F*_(2,15)_ = 1.1350, *p* = 0.3477). (D) Comparison of serum corticosterone levels among treatment groups in CFA mice (*F*_(2,12)_ = 0.6614, *p* = 0.5340).
**Figure S3:** Linalool olfactory stimulation does not induce conditioned place preference in normal male mice. (A) Representative heatmaps of travel trajectory of blank mice treated with SA or linalool odor exposure in the CPP test. (B) Summarized data for preference ratio for the odor‐paired side in normal male mice from the indicated group (*n* = 11, *t*_10_ = 0.5023, ^ns^ *p* = 0.6263) and total distance traveled (SA, *n* = 12; Linalool, *n* = 11; *F*_(1,42)_ = 3.648, ^ns^ *p* = 0.0630) in the CPP test for blank mice exposed to SA or linalool.
**Figure S4:** Linalool alleviates nociceptive hypersensitivity and induces conditioned place preference in female CFA mice. (A) The mechanical nociceptive threshold (*n* = 7, *t*_6_ = 2.520, *p = 0.0453) and thermal nociceptive threshold (*n* = 8, *t*_7_ = 6.641, **p = 0.0003) of CFA female mice treated with linalool. (B) Summarized data for preference ratio for the odor‐paired side in CFA female mice from the indicated group (*n* = 8, *t*_7_ = 2.506, *p = 0.0406) and total distance traveled (SA, *n* = 9; Linalool, *n* = 8; *F*_(1,30)_ = 2.392, ^ns^ *p* = 0.1325). (C) Representative heatmaps of travel trajectory of CFA female mice treated with SA or linalool odor exposure in the CPP test.
**Figure S5:** Linalool odor reduces anxiety‐like behavior in CFA mice. (A) Representative heatmap trajectories of mice in the open field test. (B) Summary of the percentage of time spent in the center by CFA mice (*n* = 9, *t*_8_ = 3.078, *p = 0.0152). (C) Summary of the total distance in the open area by CFA mice (*n* = 9, *t*_8_ = 2.195, ^ns^ *p* = 0.0595). (D) Representative heatmap trajectories of mice in the zero maze test. (E) Summary of the percentage of time spent in the light area by CFA mice (*n* = 11, *t*_10_ = 3.411, **p = 0.0066). (F) Summary of the number of light area entries by CFA mice (*n* = 11, *t*_10_ = 2.701, *p = 0.0223). (G) Summary of the total distance by CFA mice (*n* = 11, *t*_10_ = 1.886, ^ns^ *p* = 0.0886).
**Figure S6:** Chemogenetic activation of LH neurons alleviates pain hypersensitivity in CFA mice. (A) Experimental timeline showing viral injection, CFA induction, and subsequent pain threshold testing. (B) Schematic of hM3D(Gq)‐EGFP viral expression in LH neurons. (C) Representative image of LH neurons expressing hM3D(Gq)‐EGFP (left) and example traces of action potentials recorded before and after CNO (25 μM) application (right). (D) CNO significantly increased the firing frequency of LH neurons (*n* = 6, *F*_(1,72)_ = 75.20, *p* < 0.0001). (E) CNO (1 mg/kg) or linalool odor exposure significantly increased the mechanical withdrawal threshold (*n* = 13; *F*_(2,36)_ = 19.45, *p* < 0.0001) and the thermal withdrawal latency (*n* = 11; *F*_(2,30)_ = 6.294, *p* = 0.0052) in CFA mice.
**Figure S7:** Validation of Tet‐off activity‐dependent labeling using c‐Fos colocalization analysis in the LH.
**Figure S8:** Inputs of LH neurons. (A) Schematic showing that viral strategy for identifying the presynaptic inputs to LH. (B) Histological verification for injection of LH. (C) Representative image of EGFP signals in the AON, OT, and Piri region.
**Figure S9:** Tet‐off labeling combined with retrograde tracing confirms excitatory projections from Piri^Glu^ neurons to the LH^orexin A^. (A) Schematic illustration of the viral strategy used for activity‐dependent labeling of Piri neurons projecting to the LH^orexin A^. (B) Representative images of EGFP‐labeled input neurons in the Piri. (C, D) Representative images of the colocalization of EGFP‐labeled LH^orexin A^‐projecting Piri neurons with glutamate immunofluorescence and summarized data (*n* = 4 mice).
**Figure S10:** Outputs of Piri^Glu^ neurons. (A) Schematic of viral injection strategy in the Piri of Vglut2‐cre mice. (B) Histological verification for injection of Piri. (C) Representative image of mRuby signals in the LH, OT, and AON region.
**Figure S11:** Control experiments for evaluating potential CNO‐related off‐target effects. (A) Schematic illustration of viral strategies for control manipulation of Piri^Glu^ neurons projecting to the LH. (B) CNO administration did not affect linalool‐induced changes in mechanical withdrawal threshold (left; *n* = 12, *t*_11_ = 1.361, ^ns^ *p* = 0.2008) and thermal withdrawal latency (right; *t*_9_ = 0.3260, ^ns^ *p* = 0.7518) in control mice. (C) Schematic illustration of viral strategies for control manipulation of LH^orexin A^ neurons receiving inputs from Piri. (D) CNO administration did not affect linalool‐induced changes in mechanical withdrawal threshold (left; *n* = 14, *t*_13_ = 0.5416, ^ns^ *p* = 0.5973) and thermal withdrawal latency (right; *n* = 7, *t*_6_ = 0.2652, ^ns^ *p* = 0.7997) in control mice.
**Table S1:** Extended statistical information for Figures 1–6.

## Data Availability

All data supporting the findings of this study are present in Table S1. Additional datasets are available from the corresponding author upon reasonable request.
