# Supplementary material for: An Olfactory Piriform Cortex‐Lateral Hypothalamus Neural Circuit Contributes to Linalool‐Induced Analgesia
Source: CNS Neurosci Ther. 2026 Aug 4;32(8):e71072. doi: 10.1002/cns.71072 (PMC13435369; doi:10.1002/cns.71072)
Supplement: Supplementary file 1 [file CNS-32-e71072-s001.docx]

**Supporting Information**

**Table of Contents**

**Figure S1** Validation of the CFA-induced inflammatory pain model and screening of odorant-induced analgesic effects

**Figure S2** Effects of linalool exposure on corticosterone levels and olfactory-dependent analgesia in CFA mice

**Figure S3** Linalool olfactory stimulation does not induce conditioned place preference in normal male mice

**Figure S4** Linalool alleviates nociceptive hypersensitivity and induces conditioned place preference in female CFA mice

**Figure S5** Linalool odor reduces anxiety-like behavior in CFA mice

**Figure S6** Chemogenetic activation of LH neurons alleviates pain hypersensitivity in CFA mice

**Figure S7** Validation of Tet-off activity-dependent labeling using c-Fos colocalization analysis in the LH

**Figure S8** Inputs of LH neurons

**Figure S9** Tet-off labeling combined with retrograde tracing confirms excitatory projections from Piri^Glu^ neurons to the LH^orexin A^

**Figure S10** Outputs of Piri^Glu^ neurons

**Figure S11** Control experiments for evaluating potential CNO-related off-target effects

**Table S1** Extended statistical information for Figure 1 to Figure 6

**Figure S1** **Validation of the CFA-induced inflammatory pain model and screening of odorant-induced analgesic effects**

(A). CFA-induced changes in pain thresholds based on Electronic von Frey tests (saline, n=7, CFA, n=8, F_(3,52)_=7.792, p=0.0002).

(B). CFA-induced changes in pain thresholds based on Hargreaves tests (B, saline, n=8, CFA, n=8, F_(3,56)_=13.19, p<0.0001).

(C). Changes in mechanical pain threshold in CFA mice after exposure to different odors (n=6).

(D). Changes in thermal pain threshold in CFA mice after exposure to different odors (n=7).

**Figure S2 Effects of linalool exposure on corticosterone levels and olfactory-dependent analgesia in CFA mice**

(A). Effects of olfactory deprivation on linalool-induced changes in mechanical paw withdrawal threshold (left, n=7, t_6_=5.299, **p=0.0018) and thermal withdrawal latency (right, n=9, t_8_=2.833, *p=0.0220).

(B). Comparison of serum IL-1β levels among treatment groups in CFA mice (F_(2, 18)_=0.1433, p=0.8675).

(C). Comparison of serum TNF-α levels among treatment groups in CFA mice (F_(2, 15)_=1.1350, p=0.3477).

(D). Comparison of serum corticosterone levels among treatment groups in CFA mice (F_(2, 12)_=0.6614, p=0.5340).

**Figure S3 Linalool olfactory stimulation does not induce conditioned place preference in normal male mice**

(A). Representative heatmaps of travel trajectory of blank mice treated with SA or linalool odor exposure in the CPP test.

(B). Summarized data for preference ratio for the odor-paired side in normal male mice from the indicated group (n=11, t_10_=0.5023, ns. p=0.6263) and total distance traveled (SA, n=12; Linalool, n=11; F_(1,42)_=3.648, ns, p=0.0630) in the CPP test for blank mice exposed to SA or linalool.

**Figure S4** **Linalool alleviates nociceptive hypersensitivity and induces conditioned place preference in female CFA mice**

(A). The mechanical nociceptive threshold (n=7, t_6_=2.520, *p=0.0453) and thermal nociceptive threshold (n=8, t_7_=6.641, **p=0.0003) of CFA female mice treated with linalool.

(B). Summarized data for preference ratio for the odor-paired side in CFA female mice from the indicated group (n=8, t_7_=2.506, *p=0.0406) and total distance traveled (SA, n=9; Linalool, n=8; F_(1,30)_=2.392, ns, p=0.1325)

(C). Representative heatmaps of travel trajectory of CFA female mice treated with SA or linalool odor exposure in the CPP test.

.

**Figure S5 Linalool odor reduces anxiety-like behavior in CFA mice**

(A). Representative heatmap trajectories of mice in the open field test.

(B). Summary of the percentage of time spent in the center by CFA mice (n=9, t_8_=3.078, *p=0.0152)

(C). Summary of the total distance in the open area by CFA mice (n=9, t_8_=2.195, ns. p=0.0595).

(D). Representative heatmap trajectories of mice in the zero maze test.

(E). Summary of the percentage of time spent in the light area by CFA mice (n=11, t_10_=3.411, **p=0.0066).

(F). Summary of the number of light area entries by CFA mice (n=11, t_10_=2.701, *p=0.0223).

(G). Summary of the total distance by CFA mice (n=11, t_10_=1.886, ns. p=0.0886).

**Figure S6 Chemogenetic activation of LH neurons alleviates pain hypersensitivity in CFA mice**

(A). Experimental timeline showing viral injection, CFA induction, and subsequent pain threshold testing.

(B). Schematic of hM3D(Gq)-EGFP viral expression in LH neurons.

(C). Representative image of LH neurons expressing hM3D(Gq)-EGFP (left) and example traces of action potentials recorded before and after CNO (25 μM) application (right).

(D). CNO significantly increased the firing frequency of LH neurons (n=6, F_(1,72)_=75.20, p<0.0001).

(E). CNO (1 mg/kg) or linalool odor exposure significantly increased the mechanical withdrawal threshold (n = 13; F_(2,36)_ = 19.45, p < 0.0001) and the thermal withdrawal latency (n = 11; F_(2,30)_ = 6.294, p = 0.0052) in CFA mice.

**Figure S7** Validation of Tet-off activity-dependent labeling using c-Fos colocalization analysis in the LH

**Figure S8 Inputs of LH neurons**

(A). Schematic showing that viral strategy for identifying the presynaptic inputs to LH.

(B). Histological verification for injection of LH.

(C). Representative image of EGFP signals in the AON, OT, and Piri region.

**Figure S9 Tet-off labeling combined with retrograde tracing confirms excitatory projections from Piri^Glu^ neurons to the LH^orexin A^**

(A). Schematic illustration of the viral strategy used for activity-dependent labeling of Piri neurons projecting to the LH^orexin A^.

(B). Representative images of EGFP-labelled input neurons in the Piri.

(C and D). Representative images of the colocalization of EGFP-labelled LH^orexin A^-projecting Piri neurons with glutamate immunofluorescence and summarized data (n=4 mice).

**Figure S10 Outputs of Piri^Glu^ neurons**

(A). Schematic of viral injection strategy in the Piri of Vglut2-cre mice.

(B). Histological verification for injection of Piri.

(C). Representative image of mRuby signals in the LH, OT, and AON region

**Figure S11 Control experiments for evaluating potential CNO-related off-target effects**

(A). Schematic illustration of viral strategies for control manipulation of Piri^Glu^ neurons projecting to the LH.

(B). CNO administration did not affect linalool-induced changes in mechanical withdrawal threshold (left; n=12, t_11_=1.361, ns. p=0.2008) and thermal withdrawal latency (right; t_9_=0.3260, ns. p=0.7518) in control mice.

(C). Schematic illustration of viral strategies for control manipulation of LH^orexin A^ neurons receiving inputs from Piri.

(D). CNO administration did not affect linalool-induced changes in mechanical withdrawal threshold (left; n=14, t_13_=0.5416, ns. p=0.5973) and thermal withdrawal latency (right; n=7, t_6_=0.2652, ns. p=0.7997) in control mice.

**Table S1. Extended statistical information for Figure 1 to Figure 6**

| **Figure Panel** | **n/group** | **Primary statistic** | **Post-hoc test** | **Comparison** | **p value** | **F/t statistic** |
| --- | --- | --- | --- | --- | --- | --- |
| Fig. 1A | n=7 mice | ordinary one-way ANOVA | Dunnett's multiple comparison | Main effect of group | 0.0002 | F_(4,30)_=7.981 |
|  |  |  |  | SA vs. Pethidine | 0.0013 |  |
|  |  |  |  | SA vs. 25% | 0.9987 |  |
|  |  |  |  | SA vs. 50% | 0.1155 |  |
|  |  |  |  | SA vs. 100% | 0.0030 |  |
| Fig. 1B | n=6 mice | ordinary one-way ANOVA | Dunnett's multiple comparison | Main effect of group | 0.0045 | F_(4,25)_=4.948 |
|  |  |  |  | SA vs. Pethidine | 0.0030 |  |
|  |  |  |  | SA vs. 25% | 0.1043 |  |
|  |  |  |  | SA vs. 50% | 0.0296 |  |
|  |  |  |  | SA vs. 100% | 0.0028 |  |
| Fig. 1E | n=10 mice | two-way paired t test | - | SA vs. Linalool | 0.0183 | t_9_=2.877 |
| Fig. 1F | n=10 mice | ordinary two-way ANOVA | Šídák's multiple comparisons test | group × time interaction | 0.0491 | F_(1,40)_=4.118 |
|  |  |  |  | pre vs. post in SA | 0.3403 |  |
|  |  |  |  | pre vs. post in Linalool | 0.2500 |  |
| Fig. 2A | n=4 mice, 4 sections per mouse | two-way unpaired t test | - | SA vs. Linalool | 0.0304 | t_6_=2.820 |
| Fig. 2F-left | n=14 mice | two-way paired t test | - | SA vs. Linalool | 0.0342 | t_13_=2.366 |
| Fig. 2F-right | n=14 mice | two-way paired t test | - | SA vs. Linalool | 0.0209 | t_13_=2.627 |
| Fig. 3E-left | n=13 mice | two-way paired t test | - | SA vs. Linalool | 0.0018 | t_12_=3.994 |
| Fig. 3E-right | n=12 mice | two-way paired t test | - | SA vs. Linalool | 0.0257 | t_11_=2.577 |
| Fig. 3F-left | n=9 mice | two-way paired t test | - | SA vs. Linalool | 0.8844 | t_8_=0.1501 |
| Fig. 3F-right | n=10 mice | two-way paired t test | - | SA vs. Linalool | 0.6599 | t_9_=0.4550 |
| Fig. 4J | n=7 cells from four mice | ordinary one-way ANOVA | Šídák's multiple comparisons test | Main effect of group | 0.0007 | F_(2,18)_=11.19 |
|  |  |  |  | ACSF vs. TTX | 0.0011 |  |
|  |  |  |  | TTX+4-AP vs. TTX | 0.0017 |  |
| Fig. 4K | n=7 cells from four mice | two-way paired t test | - | ACSF vs. CNQX | 0.0010 | t_6_=5.986 |
| Fig. 5D-left | n=10 mice | two-way paired t test | - | SA vs. Linalool | 0.0050 | t_9_=3.688 |
| Fig. 5D-right | n=10 mice | two-way paired t test | - | SA vs. Linalool | 0.0031 | t_9_=4.011 |
| Fig. 5H-left | n=12 mice | two-way paired t test | - | Light-OFF vs. Light-ON | 0.0319 | t_11_=2.455 |
| Fig. 5H-right | n=12 mice | two-way paired t test | - | Light-OFF vs. Light-ON | 0.0036 | t_11_=3.681 |
| Fig. 6B-left | n=12 mice | two-way paired t test | - | vehicle vs. CNO | 0.0015 | t_11_=4.182 |
| Fig. 6B-right | n=12 mice | two-way paired t test | - | vehicle vs. CNO | 0.0148 | t_11_=2.886 |
| Fig. 6E-left | n=13 mice | two-way paired t test | - | vehicle vs. CNO | 0.0078 | t_12_=3.190 |
| Fig. 6E-right | n=12 mice | two-way paired t test | - | vehicle vs. CNO | 0.0098 | t_11_=3.115 |
| Fig. 6H-left | n=11 mice | two-way paired t test | - | vehicle vs. CNO | 0.0094 | t_10_=3.207 |
| Fig. 6H-right | n=14 mice | two-way paired t test | - | vehicle vs. CNO | 0.0044 | t_13_=3.439 |
